# The influence of disordered eating and social media’s portrayals of pregnancy on young women’s attitudes toward pregnancy

**DOI:** 10.1186/s12905-023-02177-7

**Published:** 2023-01-27

**Authors:** A. Hope Gibson, Yuliana Zaikman

**Affiliations:** Department of Psychology and Sociology, Texas A&M University – Corpus Christi, 6300 Ocean Dr., Corpus Christi, TX 78412 USA

**Keywords:** Disordered eating, Pregnancy, Unrealistic, Social media, Young women

## Abstract

**Background:**

Given the heightened emphasis on physical appearance and the prevalence of social media in young women, they are particularly vulnerable to experiencing negative body image and disordered eating. Therefore, modified social media portrayals of pregnancy could cause young women to have negative attitudes toward a potential pregnancy and subsequently not properly utilize care and resources. The present study examined the influence of disordered eating and modified portrayals of pregnancy on young women’s attitudes toward a potential pregnancy and various feelings associated with pregnancy.

**Methods:**

The sample consisted of 154 women aged 18–30, who were given the Eating-Attitudes Test-26, randomly shown either modified or unmodified social media portrayals of pregnancy, then given the Attitudes Toward Potential Pregnancy Scale and the Gestational Weight Gain Psychosocial Risk Assessment Tool.

**Results:**

A series of hierarchal regressions revealed that there were no significant main effects or interactions for young women’s attitudes toward potential pregnancy. However, women who viewed modified portrayals of pregnancy had higher self-efficacy, and women with higher levels of disordered eating had lower self-efficacy, more positive attitudes toward gestational weight gain, and lower current body image satisfaction.

**Conclusions:**

These results highlight the myriad of different attitudes that young women have toward a potential pregnancy and how these attitudes are influenced by disordered eating and social media. Our findings can be used for educating caregivers and implementing intervention strategies for women.

**Supplementary Information:**

The online version contains supplementary material available at 10.1186/s12905-023-02177-7.

## Plain English summary

Young women are more likely to have a negative body image and an unhealthy relationship with food because they are more focused on their physical appearance, especially with the rise of social media. If young women with these struggles see edited representations of pregnancy, they can develop a negative attitude about becoming pregnant in the future. This study examined how unhealthy eating habits and highly edited pictures of pregnant women impact young women’s attitudes toward a potential pregnancy. Our participants answered questions designed to see if they possess unhealthy eating thoughts and behaviors, then they were randomly shown either highly edited or not highly edited pictures of pregnant women from social media. Then, they answered questions about their attitude toward a potential pregnancy. We found that women with particularly unhealthy relationships with food were less confident about maintaining healthy eating habits throughout pregnancy. However, we also found that women who viewed the highly edited pictures were more confident about maintaining healthy eating habits throughout pregnancy. We can use these results to educate caregivers and help women get better care.

**Supplementary Information:**

The online version contains supplementary material available at 10.1186/s12905-023-02177-7.

## Background

Individuals who are highly concerned with their weight-related appearance and/or preoccupied with thoughts of eating are at-risk for developing harmful behaviors to control their eating. Such patterns of attitudes and behaviors are referred to as disordered eating (DE) [1]. DE attitudes and their subsequent behaviors range from affective changes, such as becoming irritable when hungry, to abusing food by binge eating and purging for psychological compensation [2]. While the frequency and severity of these behaviors often do not meet the criteria for a clinical eating disorder, the harmful nature of these behaviors makes them a necessary focus of further research.

Young women (between the ages of 18 and 30) are especially vulnerable to experiencing higher levels of DE behaviors and are at high risk of developing a clinical eating disorder [3]. Additionally, people in their late teens and twenties are particularly active on social media, and young women tend to engage in social comparison and self-objectification more often than young men [4]. Given these vulnerabilities, a more frequent social comparison and self-objectification would be especially harmful for young women with an existing unhealthy relationship with food.

Young women have reported varying feelings toward the possibility of a potential pregnancy, ranging from wanting to become pregnant to wanting to avoid pregnancy [5]. While there is no doubt that age and timing are the main reasons that young women avoid pregnancy, there could be other contributing factors, such as DE. The proposed study examined the influence of DE and social media portrayals of pregnancy on young women’s attitudes toward potential pregnancy.

### Disordered eating

Clinical eating disorders, such as anorexia nervosa and bulimia nervosa, affect about 5% of the general population [6]. However, there is another group of people, that despite not meeting the criteria for a clinical eating disorder, engages in unhealthy eating behaviors [1]. These people are thought to suffer from symptoms of DE.

DE represents a variety of thoughts and behaviors that highlight a preoccupation with food, including guilt, shame, and anxiety about eating; compulsive eating; frequent fluctuations in weight; restricting food intake; and purging, eating, or over-exercising to compensate for eating [7]. The frequency and severity of these thoughts and behaviors are not as severe as they are in individuals with clinical eating disorders, but they are still of clinical concern because of the negative consequences associated with them. For example, food restriction and self-induced vomiting have serious health implications such as bone loss and gastrointestinal complications [7].

DE thoughts and behaviors typically begin to develop in adolescence, often during times of stress [3], and have been shown to persist or increase in severity if left untreated into early adulthood [8]. Unfortunately for young adults with DE tendencies, this period of time is usually marked by significant life changes. Starting college and leaving home is a common step for young adults, which can be particularly stressful [9]. Given the age of onset of DE thoughts and behaviors and their association with stress, this population is at particular risk for experiencing DE symptoms [9, 10]. Indeed, previous research has found that approximately 50% of a sample of young men and women reported engaging in some kind of DE behavior(s) [1, 9–11].

DE occurs in both men and women but tends to occur more frequently in women [6], largely due to the heightened emphasis placed on women’s physical appearance [12] to adhere to cultural standards of thinness [1]. Young American women are at an increased risk of DE behaviors, with about 64–68% being affected by DE symptoms [1]. Women in an undergraduate college sample are about three times more likely than men to suffer from DE symptoms [3], with more than 11% of college women reporting that they engage in regular binge eating [13]. Therefore, it can be expected that young women who exhibit DE thoughts and behaviors would have more negative attitudes toward any physical changes that involve deviating from the social thin ideal.

### Social media portrayals of pregnancy

Social media allows for people to edit their photos to show their most flattering selves, blurring one’s real self with a digital self [14]. Consequently, social media can contribute to low self-esteem, body image dissatisfaction, more depressive symptoms, and dietary restraint [15]. People’s tendency to selectively curate posts that portray them in a flattering way [16], can lead to the normalization of DE. DE can also be reinforced through someone’s own post(s) of themselves because the content tends to be objectifying and focuses on isolated body parts instead of their face or whole body [17]. Someone who suffers from DE is likely to internalize modified social media portrayals as the ideal, perpetuating and maintaining their symptoms. Indeed, the more social media accounts a person has, the worse their DE is likely to be [18]. This information is especially concerning because the majority of young adults report having four or more social media accounts [4]. For example, in 2021, 40% of adults said they use Instagram, 69% said they use Facebook, and 21% said they use TikTok [19].

Social media portrayals of pregnancy are also subject to substantive edits. A qualitative review of pregnancy portrayals on Instagram showed that images were often professionally looking with a spotless background, perfectly edited, and intentionally posed to expose the woman’s stomach [14]. Many images are also filtered to exclude stretch marks, cellulite, and skin discoloration [20]. Hence, it is not surprising, that pregnancy portrayals in social media, especially portrayals by *influencers* (people with large social media followings), can give an unrealistic idea of pregnancy related body changes. Influencers are especially likely to push a perfect pregnancy narrative because they often receive financial benefits from sponsors. Given that women tend to trust these sources to be truthful, such information could be harmful to women’s expectations for their pregnancy and lead to maladaptive beliefs and behaviors [14].

Social media can also be damaging to pregnant women because of social comparisons to their friends and family. Some women have described that seeing friends that are smaller than them while pregnant caused them to judge themselves and have a negative impact on their body image [20]. Social comparison theory suggests that people often evaluate their abilities based on the opinions and abilities of others [22]. According to this theory, people make upward social comparisons when they view the abilities of others to be superior to their own, which tends to lead to dissatisfaction with oneself. Therefore, women’s attitudes toward potential pregnancy can be impacted by the modified images of pregnant women on social media.

### Attitudes toward potential pregnancy

Often, pregnancies are discussed in terms of intended or unintended. However, women’s attitudes toward potential pregnancies are much more complex than just intending to be or not intending to be pregnant [23]. There are additional variables that contribute to attitudes toward a potential pregnancy, such as feelings about gaining weight during pregnancy (gestational weight gain), confidence regarding one’s ability to create and maintain healthy eating habits throughout pregnancy, and pre-pregnancy weight satisfaction [24]. Given that young women are more susceptible to DE [1, 6] and are frequent consumers of social media [4], the cumulative impact of modified social media images along with DE can be detrimental. Specifically, if young women with higher levels of DE are exposed to modified images of pregnancy on social media, they could feel more negatively toward a potential pregnancy and its associated changes.

### Overview and hypotheses

The current study examined if there is a relationship between DE and young women’s attitudes toward a potential pregnancy. Additionally, the study assessed if various portrayals of pregnancy on social media influence such attitudes. Specifically, we manipulated whether participants saw modified or unmodified images of pregnancy and measured their DE thoughts and behaviors as predictor variables. We measured the effects of the predictor variables on attitudes toward a potential pregnancy, self-efficacy about maintaining healthy eating habits during pregnancy, attitudes toward gestational weight gain, and current body image when considering a potential pregnancy.

Based on previous literature, we predicted that young women with higher levels of DE (hypothesis 1), young women who viewed modified social media portrayals of pregnancy (hypothesis 2), and young women with higher levels of DE who viewed modified social media portrayals of pregnancy (hypothesis 3) would have more negative attitudes toward a potential pregnancy, have lower self-efficacy about maintaining healthy eating habits during pregnancy, have more negative attitudes toward gestational weight gain, and have lower current body image when considering a potential pregnancy.

## Methods

### Participants

Participants were recruited from psychology classes at Texas A&M University – Corpus Christi and through posts on social media. Only participants who are 18 years old or older, female, and college students were eligible to participate in the study. The initial sample consisted of 177 participants. Six participants were removed because they were male, two were removed for identifying as nonbinary, and two were removed for answering the attention check question incorrectly. Finally, as our goal was to examine younger women’s attitudes toward potential pregnancy and because previous literature considered women under 30 to be young women, 13 participants were removed because they were above the age of 30. The final sample consisted of 154 women. The majority of the participants were Hispanic (49.6%) or White (49%), heterosexual (70.9%), and moderate (22%) or liberal/very liberal (38.3%). Additionally, the majority of the participants were single (42.6%) or in a committed relationship (40.4%). Data was collected in March 2022.

### Measures

#### Eating Attitudes Test-26 (EAT-26)

The Eating Attitudes Test-26 (EAT-26), developed from the original Eating Attitudes Test-40, is a three-part questionnaire that uses body mass index (BMI), scores from a 26-item questionnaire, and four behavioral assessment questions as a screening to identify whether people are at-risk of developing an eating disorder [25]. This tool does not provide a diagnosis and is only to be used as a preliminary assessment of the presence of DE patterns of thoughts and behaviors. The bulk of the EAT-26 test consisted of 26 items divided into three subscales: dieting (13 items), bulimia and food preoccupation (6 items), and oral control (7 items). Examples of items on the EAT-26 include “I like eating with other people,” “I become anxious prior to eating,” and “I find myself preoccupied by food.” Items were rated by participants on a 5-point Likert-type scale (*always—never*). Participants’ scores were calculated by summing the points from each question, and scores at 20 or above are thought to be indicative of being at-risk for developing an eating disorder. There were additional 5 items that concerned behavioral aspects of DE. In the first four items, participants were asked how often they engage in behaviors such as binge eating and excessive exercise. Items were rated on a 6-point Likert-type scale (*never—once a day or more*). The last item was answered “yes” or “no” and concerned recent rapid weight loss. For the purpose of this study, we split the EAT-26 into two subscales that represent DE thoughts and DE behaviors. DE thoughts were measured by looking at the core 26 questions and DE behaviors were measured by the supplemental 5 behavioral questions. The reliability of DE thoughts subscale was high (0.87), while the reliability of DE behaviors subscale was low (0.51).

#### Attitudes Toward Potential Pregnancy Scale (APPS)

The Attitude Toward Potential Pregnancy Scale (APPS) is a brief self-report questionnaire that was developed to measure women’s feelings toward a potential pregnancy and their efforts to either become pregnant or avoid pregnancy [26]. The scale consists of five items that are measured on a Likert scale. The scale for each item is the same, but the wording is changed to match the item content. For example, the question “How important is it to you to avoid becoming pregnant now?” has anchors 1 and 5 as “*Not at all important*” and “*Very important*,” respectively, and the question “How worried would you be if you were pregnant now has anchors 1 and 5 as “*Not worried at all*” and “*Extremely worried*.” Scores are calculated by summing the point values of each item and scores range from 5 to 25. This measure has a Cronbach’s alpha for internal consistency of 0.86 and the item-total correlations vary from 0.56 to 0.75 [26]. The reliability of this scale was high (0.81).

#### Gestational Weight Gain Psychosocial Risk Assessment Tool

The Weight-Related Behaviors Questionnaire (WRB-Q) is a validated measure used to assess pregnancy-related factors that affect weight gain and retention during and after pregnancy [27]. The original measure contains 49 items, however we used the short-form 12 item questionnaire called the Gestational Weight Gain Psychosocial Risk Assessment Tool to look for women that are at risk for gaining excessive weight during pregnancy [24]. The adapted questionnaire was split into three subscales: Self-Efficacy, Attitudes Toward Weight Gain, and Body Image. All three subscales were measured on a Likert scale and the values were different for each group. Self-Efficacy was measured on a 5-point scale ranging from 1 (*Very Sure*) to 5 (*Very Unsure*) and Attitudes Toward Weight Gain was measured on a 5-point scale ranging from 1 (*Strongly Agree*) to 5 (*Strongly Disagree*). The first two items on the Body Image subscale were measured on a 4-point scale ranging from 0 (*Very Satisfied*) to 3 (*Very Dissatisfied*), and the other two items on the second Body Image subscale were measured on a 3-point scale ranging from 0 (*Too Heavy*) to 2 (*Too Light*). Items in the Self-Efficacy and Attitudes Toward Gestational Weight gain subscales were reverse coded so that higher scores indicate higher levels of the construct being measured. Therefore, a high score in self-efficacy indicates that the person has high self-efficacy about maintaining healthy eating habits during pregnancy. For the purposes of this study, some questions were slightly altered to be hypothetical questions about a potential pregnancy. The reliability of the subscales was high (0.85 self-efficacy, 0.91 attitudes toward gestational weight gain, and 0.85 for body image).

#### Pregnancy photos

The social media app Instagram was used to find pictures that show portrayals of pregnancy in either modified or unmodified images. After reaching out to several women on Instagram asking if they would be willing to grant us permission to use their photos in our study, two women agreed. We used six pictures from each woman for a total of 12 images. Specifically, there were three modified images and three unmodified images of each woman. Modified pictures had visual evidence of editing due to particularly smooth, blemish-free skin and staged sets. Unmodified pictures showed natural skin, such as stretch-marks and discoloration in day-to-day environments. See Additional file 1: Appendix A for sample pictures.

### Procedure

Participants were first presented with an informed consent before proceeding to the survey. All participants completed the full Eat-26 questionnaire. Next, participants were randomly assigned to view either six photos of the pregnant women that were highly modified or six photos of the pregnant women that were not highly modified. Participants were presented with one photo at a time and were able to click through the photos at their own pace. Then, all participants were given the Attitude Toward Potential Pregnancy Scale followed by the Gestational Weight Gain Risk Assessment Tool. The last step of the survey consisted of demographic questions. At the end of the survey, participants were presented with a link to an eating-disorder help and information website resource.

## Results

According to the guidelines of the EAT-26, 95 women (67.4%) had lower levels of DE, and 43 women (30.5%) had higher levels of DE and would qualify for a referral to an eating disorder specialist. A series of hierarchal regressions were used to test if the presence of DE and the presentation of either modified or unmodified social media portrayals of pregnancy significantly predicted young women’s attitudes toward potential pregnancy, perceived self-efficacy during pregnancy, attitudes toward gestational weight gain, and body image when considering a potential pregnancy. For each hierarchal regression, we entered in Step 1 pregnancy portrayal and centered versions (based on scale means) of DE thoughts and DE behaviors. In Step 2, we entered the two two-way interactions between pregnancy portrayal and DE thoughts and pregnancy portrayal and DE behaviors. The first regression examined the effects on attitudes toward potential pregnancy. No main effects or two-way interactions were observed.

The second regression examined if DE and pregnancy portrayal significantly predicted young women’s perceived self-efficacy to have healthy eating habits throughout future pregnancy. There was a main effect for pregnancy portrayal, $\beta = .188, p = .027,{ }f^{2} = .04.$ Participants who were shown modified social media portrayals of pregnancy had higher self-efficacy about having healthy eating habits during pregnancy, compared to participants who saw the unmodified images. There was another main effect for the DE behaviors, $\beta = - .25, p = .016, f^{2} = .05.$ Specifically, women who highly endorsed DE behaviors had lower self-efficacy about having healthy eating habits during pregnancy than women who endorsed to a lesser degree DE behaviors. There was no main effect for DE thoughts.

A third regression was performed to test if disordered eating and pregnancy portrayal predicted young women’s attitudes toward gestational weight gain. There was a main effect for DE thoughts, $\beta = .354, p < .001, f^{2} = .17.$ Specifically, women who had higher levels of DE thoughts had more positive attitudes about weight gain during pregnancy than women with lower levels of disordered eating thoughts.

A fourth regression examined the influence of disordered eating and pregnancy portrayal on young women’s body image when considering a future pregnancy. There was a main effect for DE thoughts, $\beta = - .41, p < .001, f^{2} = .25$. Specifically, women with higher levels of DE thoughts were less satisfied with their current body image when considering a potential pregnancy.

## Discussion

This research examined how DE and social media portrayals of pregnancy influence young women’s attitudes toward a potential pregnancy, perceived self-efficacy about maintaining healthy eating habits throughout pregnancy, attitudes toward gestational weight gain, and pre-pregnancy body image. Specifically, we hypothesized that young women with higher levels of DE thoughts and/or behaviors would have more negative attitudes toward a potential pregnancy, have lower self-efficacy about maintaining healthy eating habits throughout pregnancy, have more negative attitudes toward gestational weight gain, and have lower body image when considering a potential pregnancy. Supporting our hypothesis, young women with higher levels of DE behaviors had lower perceived self-efficacy about maintaining healthy eating habits throughout pregnancy. Pregnancy can be a time of significant stress for women, and DE behaviors tend to worsen during periods of distress [3]. Therefore, women who struggle with DE behaviors such as binging, purging, and excessive exercise pre-pregnancy anticipate that they would continue to struggle and would be unable to maintain healthy eating habits throughout a potential pregnancy.

Additionally, contrary to our hypothesis, we found that young women with higher levels of DE thoughts had more positive attitudes toward gestational weight gain. This is unexpected as we expected that women with significant preoccupations with food and eating would be less accepting of weight gain, even during pregnancy [24]. A possible explanation for this could be that young women who are preoccupied with their weight, body shape, and eating habits would not feel as pressured to avoid gaining weight if they were to become pregnant. Because gaining weight during pregnancy is necessary, young women with higher levels of DE thoughts may not be concerned with the weight gain like they normally would be and may even view pregnancy as a justified reason for the weight gain.

In addition, supporting our hypothesis, young women with higher levels of DE thoughts had lower pre-pregnancy body image when considering a potential pregnancy. We predicted this effect because young women with higher levels of DE thoughts tend to have particularly negative weight satisfaction, even without considering a potential pregnancy [12]. Taken together, hypothesis 1 was partially supported.

Given that modified pictures of pregnancy on social media can give women unrealistic expectations of what they should look like if they were to become pregnant [20], we predicted that young women who viewed modified social media portrayals of pregnancy would have more negative attitudes toward a potential pregnancy, lower self-efficacy about maintaining healthy eating habits during pregnancy, more negative attitudes toward gestational weight gain, and lower body image when considering a potential pregnancy. Interestingly, our results illustrate that young women who viewed modified social media portrayals of pregnancy were more confident in their ability to maintain healthy eating habits throughout pregnancy than women who viewed unmodified social media portrayals of pregnancy. The modified images could have influenced participants to believe that if they maintained healthy eating habits throughout pregnancy they could look like the women in the pictures. Although being influenced by social media is thought to be generally negative for young women, these results suggest that young women can be positively influenced and perhaps even empowered by portrayals of pregnancy on social media, despite them being unrealistic. Therefore, hypothesis 2 was not supported.

The third hypothesis stated that young women with higher levels of DE who viewed modified social media portrayals of pregnancy would have more negative attitudes toward a potential pregnancy, lower self-efficacy about maintaining healthy eating habits during pregnancy, more negative attitudes toward gestational weight gain, and lower current body image when considering a potential pregnancy than young women with low levels of DE who viewed unmodified social media portrayals of pregnancy. Our results found no support for the influence of DE and social media portrayal of pregnancy on young women’s attitudes toward a potential pregnancy. In fact, we found no other effects on women’s attitudes toward a potential pregnancy. It is possible that the lack of effects might be driven by the age of the women (*M* = 20.65, *SD* = 2.45). Young women in their late teens and twenties most likely do not desire or try to become pregnant because they are typically either pursuing higher education, starting a career or just do not feel prepared to have a child [5]. Therefore, it is possible that the participants in this study were more likely to be motivated to avoid a potential pregnancy for factors other than DE or the manner in which pregnancy is portrayed on social media.

Finally, this interaction was not significant for any of the other measures: self-efficacy, attitudes toward gestational weight gain, or current body image when considering a potential pregnancy. It is possible that there is no relationship between DE and social media portrayals of pregnancy. However, it is more likely that this relationship exists, but the manner in which we examined it, with a relatively short exposure to pregnancy portrayals in social media might not have had the substantial impact that we were expecting. It is more likely that continuous and prolonged exposure to the manner in which pregnancy is portrayed in social media relates to the development or maintenance of DE and its cumulative impact on self-efficacy about maintaining healthy eating habits during pregnancy, more negative attitudes toward gestational weight gain, and lower current body image when considering a potential pregnancy. Taken together, hypothesis 3 was not supported.

### Limitations and future directions

One limitation was the likely floor effect we observed regarding young women’s attitudes toward potential pregnancy. Young women from 18 to 30 years-old are less likely to desire or try to become pregnant, regardless of their level of DE, because they are focused on their education, starting their careers, or just waiting until they are older to have children [5]. Additionally, despite having an adequate number of participants, replication with a larger sample of respondents closer to the average age at first birth (in 2014 the average age of first-time mothers was 26.3, [28]) would increase the generalizability of the findings.

Another limitation that the study might have had is the images used to depict portrayals of pregnancy. We utilized the same two women for the modified and unmodified social media portrayals of pregnancy. We chose to use the same women for both categories to avoid any confounding factors using several different women. However, the two women that we used for the study were traditionally beautiful, and their unmodified pictures may not exhibit average looking pregnant women. Future research can use pictures of less traditionally attractive women so that there is more of a visual difference between the modified and unmodified pictures. Future research could also use pictures of different women for both categories instead of using the same women for both.

Additionally, future research could measure the amount of time young women spend on social media and the duration young women for which they view each picture, especially portrayals of pregnancy. Due to the short amount of time participants were exposed to social media portrayals of pregnancy, there may not have been a strong enough effect to show significant results. Therefore, future research could examine if viewing modified social media portrayals of pregnancy for extended periods of time has a greater impact on young women’s attitudes toward a potential pregnancy and other related factors.

Finally, given some of the unexpected findings, a more qualitative approach to this research could be utilized. Specifically, the relationship between possessing higher levels of DE thoughts and more positive attitudes toward gestational weight gain should be examined qualitatively. Using a qualitative analysis would allow women to share their exact feelings rather than relying on multiple choice questions with limited elaboration. A more extensive evaluation of young women’s attitudes toward potential pregnancy and body image can be used to ensure better outcomes for both mother and child [21]. Having women’s reasons for their attitudes would allow for more comprehensive care for women because healthcare professionals would have a deeper understanding of how DE and social media affects women’s attitudes toward a potential pregnancy.

## Conclusion

This study examined the influence of DE and social media portrayals of pregnancy on young women’s attitudes toward a potential pregnancy, self-efficacy about maintaining healthy eating habits throughout pregnancy, attitudes toward gestational weight gain, and current body image when considering a potential pregnancy. Our findings illustrate that DE appears to impact women’s self-efficacy, attitudes toward gestational weight gain, and general satisfaction with current body image. These findings can be used by both mental and physical health practitioners to be aware of the risks of DE and know how to incorporate such problems into perinatal care.

## Supplementary Information

**Additional file 1: Appendix A: **Modified and Unmodified Pregnancy Photos.

## Data Availability

The datasets generated during and/or analyzed during the current study are not publicly available because informed consent provisions did not cover public data sharing but are available from the corresponding author on reasonable request.

## Abbreviations

DE: Disordered eating
EAT-26: Eating Attitudes Test-26
APPS: Attitude Toward Potential Pregnancy Scale
