# Supplementary figures and images for: The influence of disordered eating and social media’s portrayals of pregnancy on young women’s attitudes toward pregnancy
Source: BMC Womens Health. 2023 Jan 27;23:38. doi: 10.1186/s12905-023-02177-7 (PMC9883907; doi:10.1186/s12905-023-02177-7)

**Appendix A**

**Modified images of pregnancy**

**
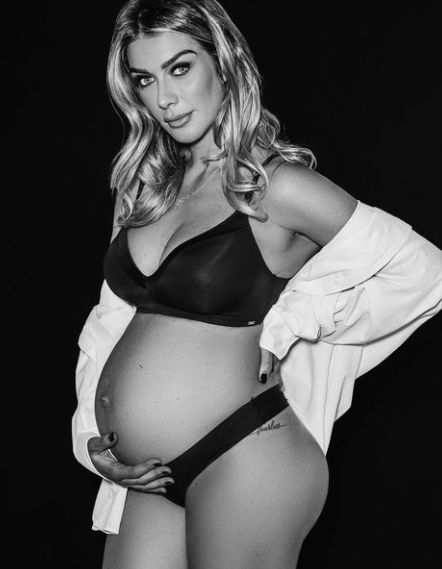

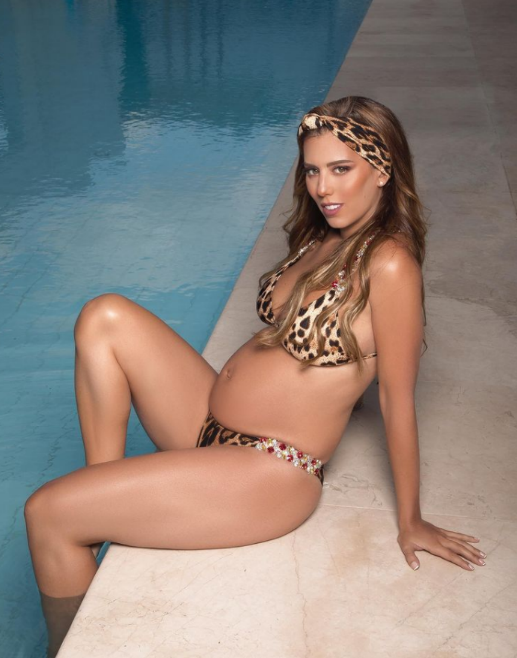
**

**Unmodified images of pregnancy**

**
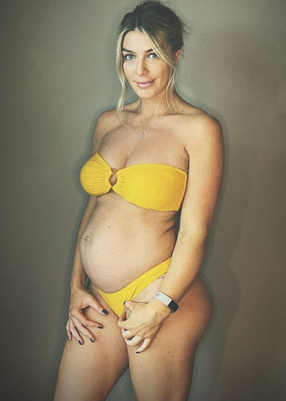

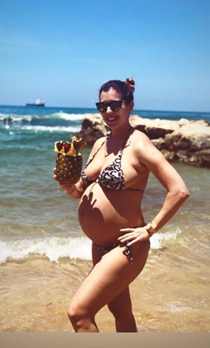
**

|  |  |
| --- | --- |

Supplement: Supplementary file 1 — Additional file 1: Appendix A: Modified and Unmodified Pregnancy Photos. [file 12905_2023_2177_MOESM1_ESM.docx]
